# Supplementary material for: Electron recombination of rotationally cold D2H+ ions
Source: Nat Commun. 2025 Aug 19;16:7738. doi: 10.1038/s41467-025-62734-6 (PMC12365165; doi:10.1038/s41467-025-62734-6)
Supplement: Supplementary file 1 — Supplementary Information [file 41467_2025_62734_MOESM1_ESM.pdf]

Online Supplemental Material

Electron recombination of rotationally cold D_2H^+ ions

A. Znotins[1], A. Faure[2], C.H. Greene[3], M. Grieser[1], F. Grussie[1], L.W. Isberner[4,1], Á. Kálosi[5,1], V. Kokoouline[6], D. Müll[1], D. Paul[5,1], M. Pezzella[7], D.W. Savin[5], S. Schippers[4], J. Tennyson[7], A. Wolf[1], O. Novotný[1], H. Kreckel[1]

[1] *Max-Planck-Institut für Kernphysik, Saupfercheckweg 1, 69117 Heidelberg, Germany*

[2] *Univ. Grenoble Alpes, CNRS, IPAG, 38000 Grenoble, France*

[3] *Department of Physics and Astronomy, Purdue University, West Lafayette, Indiana 47907, USA*

[4] *I. Physikalisches Institut, Justus-Liebig-Universität Gießen, D-35392 Gießen, Germany*

[5] *Columbia Astrophysics Laboratory, Columbia University, New York, NY 10027, USA*

[6] *Department of Physics, University of Central Florida, Orlando, FL 32816, USA*

[7] *Department of Physics and Astronomy, University College London, London WC1E 6BT, United Kingdom*

In this Supplemental Material we present an outline of the cross section calculations for inelastic electron collisions of D_2H^+, followed by the description of our updated theoretical calculations for the dissociative recombination (DR) of energetically low-lying rotational states. Finally, we derive kinetic temperature DR rate coefficients for D_2H^+, based on the experimental data.

Contents

1 Cross section calculations for inelastic electron collisions **2**

2 Updated DR calculations for D_2H^+ **4**

3 Kinetic temperature DR rate coefficient for D_2H^+ **9**

1 Cross section calculations for inelastic electron collisions

The cross sections for electron-impact rotational excitation $\sigma_{J_\tau \to J'_{\tau'}}(E)$ as a function of collision energy E were computed using the R-matrix approach combined with the fixed-nuclei (FN) and Coulomb-Born (CB) approximations. The fixed-nuclei reactance matrices (K-matrices) were obtained using the UK molecular R-matrix code (UKRMol+) [1] run with the Quantemol Electron Collision (QEC) expert system [2]. The R-matrix calculations were performed within the C_{2v} point group for the ground-state electronic configuration of H_3^+ at its equilibrium geometry. A static exchange plus polarization (SEP) calculation was performed using an R-matrix boundary of 10 a_0 which considered energies up to 15 eV. The target was represented using a cc-pVDZ Gaussian basis set. The dipole moment used for D_2H^+ is obtained geometrically from the difference between the center-of-mass and center-of-charge for the isotopologue.

Working in C_{2v} symmetry, each R-matrix calculation produced four fixed-nuclei K-matrices (for the four symmetries A_1, B_1, B_2 and A_2) which were processed by the `POLYDCS` code [3] to generate rotational excitation cross sections $\sigma^{\mathrm{FN}}(J_\tau \to J'_{\tau'})$, where $\tau = K_a - K_c$ is the pseudo-quantum number that varies from $-J$ to J. We note that the rotational eigenfunctions and eigenenergies of D_2H^+ were computed with a new, modern FORTRAN90 version of the `ASYMTOP` code [4], with consistent eigenvectors in agreement with the tests of [5] for all representations.

Owing to the limited number of partial waves included in the K-matrices ($l \leq 4$), the `POLYDCS` cross sections were complemented with the CB theory (for high-partial waves $l > 4$), following the method described in [6] in which the final cross section is the sum of the FN cross section and the difference between the total (σ^{TCB}) and partial (σ^{PCB}) CB cross sections:

$$\sigma(J_\tau \to J'_{\tau'}) = \sigma^{\mathrm{FN}}(J_\tau \to J'_{\tau'}) + \sigma^{\mathrm{TCB}}(J_\tau \to J'_{\tau'}) - \sigma^{\mathrm{PCB}}(J_\tau \to J'_{\tau'}). \tag{S1}$$

This CB completion procedure was applied to dipole-allowed transitions (here $\Delta J = 0, \pm 1$) only. High-partial wave effects were shown to be negligible for dipole-forbidden transitions in the case of linear molecular ions [7]. Because the dipolar CB approximation was implemented here for the first time for an asymmetric-top ion, we provide below the two useful equations.

Following previous derivations of the CB approximation [6, 8, 9], the total CB cross section for a dipole-allowed rotational excitation $J_\tau \to J'_{\tau'}$ can be reformulated using the Einstein $A(J'_{\tau'} \to J_\tau)$ coefficients as follows (adopting Gaussian units):

$$\sigma^{\mathrm{TCB}}(J_\tau \to J'_{\tau'}) = \left(\frac{3}{4\pi^2}\right) \frac{\pi}{k_i^2} A(J'_{\tau'} \to J_\tau) \left(\frac{3hc^3}{64\pi^4 \nu^3}\right) \left(\frac{1}{e^2 a_0^2}\right) \frac{(2J'+1)}{(2J+1)} f_{E_1}(\eta, \zeta), \tag{S2}$$

where k_i (k_f) is the initial (final) wave number of the electron, $A(J'_{\tau'} \to J_\tau)$ the Einstein coefficient of the rotational transition (extracted from the Cologne Database for Molecular Spectroscopy[1]), h is the Planck's constant, c is the speed of light, ν is the frequency of the transition, e is the elementary charge, a_0 is the Bohr radius, and $f_{E1}(\eta,\zeta)$ is a function related to the $E1$ nuclear Coulomb function, with $\eta = -1/k_i$ and $\zeta = 1/k_i - 1/k_f$. The exact expression of $f_{E1}(\eta,\zeta)$ can be found in Eq. (22) of Ref. [8]. Similarly , the partial CB cross section was implemented as:

$$\sigma^{\rm PCB}(J\tau \to J'_{\tau'}) = \left(\frac{16\pi}{3}\right)\left(\frac{k_f}{k_i}\right) A(J'_{\tau'} \to J_\tau)\left(\frac{3hc^3}{64\pi^4\nu^3}\right)\left(\frac{1}{e^2a_0^2}\right)\frac{(2J'+1)}{(2J+1)}\sum_{l_i,l_f}(2l_i{+}1)(2l_f{+}1)\begin{pmatrix} l_i & l_f & 1 \\ 0 & 0 & 0 \end{pmatrix}^2 |M^{-2}_{l_il_f}|^2, \tag{S3}$$

where the radial matrix elements $M^{-2}_{l_il_f}$ are given in Eq. (A2) of [6].

It should be noted that the validity of the FN approximation was previously tested on H_3^+ by comparing FN cross sections with calculations based on the MQDT rotational frame transformation method [10]. The FN cross sections were found to be accurate down to threshold, except in the presence of large resonances occuring for transitions with $\Delta J = 1$. In general, however, the FN approximation was shown to be quite

[1]https://cdms.astro.uni-koeln.de/

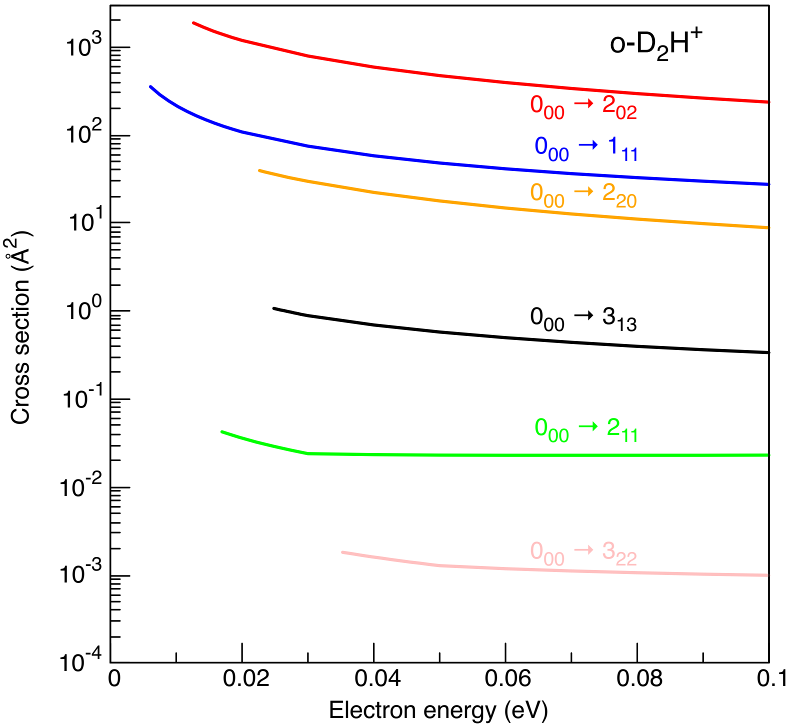

Supplementary Figure 1: Rotational excitation cross sections for ortho-D_2H^+ ions in their ground state 0_{00}, as a function of the electron energy.

successful. Moreover, the accuracy of the CB completion was confirmed experimentally recently in the case of CH^+ [11, 12].

In practice, the final excitation cross sections (Eq. S1) for D_2H^+ were obtained for electron energies in the range $0.01 - 0.5$ eV and were then extrapolated as $1/E$ down to threshold. All transitions involving D_2H^+ rotational levels with $J \leq 3$ were considered. The CB correction was found to increase the dipolar cross sections by a factor of $2 - 3$. On the other hand, because the dipole of the deuterated ions is moderate (< 1 D), the largest excitation cross sections were found for dipole-forbidden transitions with $\Delta J = 2$, as illustrated in Supplementary Figure 1 for excitations from the ground state $J_{K_a K_c} = 0_{00}$ of o-D_2H^+. We note that transitions with even $\Delta\tau$ are allowed in D_2H^+. These selection rules reflect the conservation of the nuclear-spin symmetry (ortho or para) of the nuclear-spin isomer.

These inelastic cross sections were folded with the experimental velocity distribution and included in the master equation model. They were also convolved with Maxwell-Boltzmann distributions to provide kinetic temperature state-to-state rate coefficients for astrochemical modelling of D_2H^+ excitation and emission. These rate coefficients will be made available through the EMAA database https://dx.doi.org/10.17178/EMAA.

2 Updated DR calculations for D_2H^+

The present theoretical approach for modeling dissociative recombination of D_2H^+ follows the treatment discussed by Kokoouline and Greene [13] with some changes in the way how the channel functions for the electron-ion scattering are constructed.

2.1 Channel wave functions for the e^--D_2H^+ system

In the present approach, the DR cross section is computed using the scattering matrix $\mathcal{S}$ for electron-ion scattering. Elements $\mathcal{S}_{i',i}$ of the matrix represent scattering from channel i to channel i' of the ion. The index of channel i refers for the quantum state of the ion (before or after a collision) and also to the partial wave of the incident electron. We use the reference frame in which the total angular momentum (quantum number N) and its laboratory projection m of the electron-ion system are well-defined. As a result, the scattering matrix $\mathcal{S}$ is diagonal over N. During an electron-ion collision, the symmetry (using the more precise terminology, the irreducible representation - irrep) Γ of the total wave function with respect to symmetry operators of the C_{2v} group is conserved. The projection of the angular momentum on a laboratory axis is also conserved. In addition, in the treatment, we use only p-wave scattering. It was shown that it is the major contributor to the DR probability. Accounting for the s-wave and d-scattering is left for a future publication.

Constructing the channel functions, the Born-Oppenheimer approximation is used: Channel functions are

Operators: Irreps	E	(12)	E^*	$(12)^*$
A_1	1	1	1	1
A_2	1	1	-1	-1
B_1	1	-1	-1	1
B_2	1	-1	1	-1

Supplementary Table 1: Operators, characters, and irreps of C_{2v} symmetry group.

the product of vibrational, rotational, electronic (partial wave of the incident electron), and nuclear spin factors,

$$\Phi_{\text{total}} = \Phi_{\text{vib}}\Phi_{\text{rot}}\Phi_{\text{el}}\Phi_{\text{ns}} \,. \tag{S4}$$

We stress here that the Born-Oppenheimer approximation is used only for channel functions, the incident electron couples different channels, so that the total wave function of the system is a linear combination of the channel functions during and after a collision. In principle, a more accurate treatment could account for channel functions by relaxing the Born-Oppenheimer approximation, which will be the subject of future work. The channel functions are enumerated by an index made of vibrational, rotational, nuclear spin, partial-wave numbers, and the index Γ of irrep.

The scattering matrix is initially constructed in the molecular reference frame (MF) and then transformed to the laboratory frame (LF) using a frame transformation. The channel functions in the two reference frames are different but they have the same general structure of Eq. (S4). Therefore, the following elements are needed to construct the scattering matrix: vibrational, rotational, nuclear-spin functions in the two reference frames (partial-wave functions are just spherical harmonics), the scattering matrix in the MF, and the matrix of transformation between the two frames. Below we discuss briefly how the rotational, vibrational, electronic, and nuclear-spin functions are constructed. Also, the symmetry of the wave function is reviewed: The channel functions of Eq. (S4) should be symmetric under exchange of two identical bosonic nuclei in D_2H^+.

The C_{2v} symmetry group has four operators. We use here the notations by Bunker and Jensen [14]. The operators are E, (12), E^*, and $(12)^*$, which can be obtained by two operators only: inversion E^* and permutation (12) of two identical (D) particles.

Supplementary Table 1 summarizes irreps of the C_{2v} group and gives characters of the group, which, in this case, determine whether a state of a given irrep changes sign or stays the same under an operator. Possible irreps of the total channel function Φ_{total} of D_2H^+ are A_1 or A_2. Supplementary Table 2 gives the correspondence between the operators of the C_{2v} group and the operations acting on particular factors of the total wave function in Eq. (S4). The orientation of the coordinate axes in the molecular frame is the same as in Ref. [15]: The z-axis is orthogonal to the plane of the molecule and the x-axis is the axis of the C_{2v} symmetry. Similarly to

Operators	E	(12)	E^*	$(12)^*$
Vibrational	E	C_{2x}	σ_{xy}	σ_{xz}
Rotation	E	R_x^π	R_z^π	R_y^π
Electronic	E	C_{2x}	σ_{xy}	σ_{xz}
Nuclear spin	E	ρ_{12}	E	ρ_{12}

Supplementary Table 2: The table summarizes the transformations of vibrational, rotational, electronic, and nuclear spin wave functions under the C_{2v} operators. R_a^α is a rotation through the angle α about the axis a. The operator C_{2x} is the operator of rotation about the x axis, the operators of the type σ_{xy} are reflections through the xy plane. The operator ρ_{12} exchanges identical nuclei.

Γ	p_1	p_2
A_1	1	1
A_2	-1	1
B_1	-1	-1
A_1	1	-1

.

Supplementary Table 3: Correspondence between p_1, p_2 and the C_{2v} irreducible representations

Ref. [13], for characterization of the different factors it is convenient to introduce additional quantum numbers – eigenvalues $p_1 = \pm 1$ of the inversion operator E^* and $p_2 = \pm 1$ of operator (12). Each factor in Eq. (S4) has both quantum numbers well-defined. The combination of the two quantum numbers corresponds to one and only one irrep of the group. Supplementary Table 3 provides the correspondence between the quantum numbers p_1 and p_2 and irreps of C_{2v}

2.2 Vibrational wave functions

Due to the plane geometry of the D_2H^+ ions, the vibrational functions Φ_v are unchanged after E^*

$$E^* \Phi_v = \sigma_{xy} \Phi_v = \Phi_v \tag{S5}$$

and, therefore, can be only of A_1 and B_2 irreps. The vibrational functions are calculated as discussed in Ref. [13]. Here, we only give expressions for principal moments of inertia, needed to compute the rotational constants for each vibrational state. These are given by Johnson [16] in terms of the hyperradius R and the hyperangle θ,

$$\begin{aligned} I_x &= \frac{1}{2}\mu R^2(1-\sin\theta), \\ I_y &= \frac{1}{2}\mu R^2(1+\sin\theta), \\ I_z &= I_x + I_y = \mu R^2\,, \end{aligned} \tag{S6}$$

where μ is the reduced three-body mass. The rotational constants are calculated as expectation values

$$B_i = \langle \Phi_v | 1/(2I_i) | \Phi_v \rangle, \tag{S7}$$

where the integral is evaluated over the three hyperspherical coordinates.

2.3 Rotational wave functions

The rotational functions in Eq. (S4) are constructed in a similar way in MF and LF. They are obtained solving the eigenvalue problem for the asymmetric top rotator. The rotational Hamiltonian has the form [14]:

$$H_{rot} = \frac{B_x + B_y}{2}\hat{N}^2 + \left(B_z - \frac{B_x + B_y}{2}\right)\hat{N}_z^2 \tag{S8}$$

$$+\frac{B_x - B_y}{4}\left(\hat{N}_+^2 + \hat{N}_-^2\right), \tag{S9}$$

where $\hat{N}_\pm = \hat{N}_x \pm i\hat{N}_y$, $\hat{N}_x, \hat{N}_y, \hat{N}_z$ are operators of different components of the angular momentum of the molecule (as a rigid rotor) and $\hat{N}^2 = \hat{N}_x^2 + \hat{N}_y^2 + \hat{N}_z^2$. $B_i (i = x, y, z)$ are the rotational constants for rotation about the corresponding axis. For geometries different than the C_{2v} geometry (such as equilibrium of the D_2H^+ ions), the axes x and y are defined by principal axes of inertia. The values of the rotational constants depend on the vibrational state of the channel function and are calculated numerically with Eq. S7. Note that with this choice of axes, the values of the rotational constants for the lowest vibrational levels satisfy $B_y > B_x > B_z$ for D_2H^+. The rotational Hamiltonian is represented as a matrix in the basis of symmetric-top functions

$$\mathcal{R}_{km}^N(\alpha, \beta, \gamma) = \left[\frac{2N+1}{8\pi^2}\right]^{1/2} \left[D_{m,K}^N(\alpha, \beta, \gamma)\right]^*. \tag{S10}$$

Matrix elements of H_{rot} are given, for example, by Bunker and Jensen [14]. The only non-zero elements are

$$\langle N, k, m | \hat{N}^2 | N, k, m \rangle = N(N+1),$$
$$\langle N, k, m | \hat{N}_z^2 | N, k, m \rangle = k^2,$$
$$\langle N, k-2, m | \hat{N}_+^2 | N, k, m \rangle = \langle N, k, m | \hat{N}_-^2 | N, k-2, m \rangle =$$
$$\left([N(N+1) - (k-1)(k-2)] \left[N(N+1) - k(k-1) \right] \right)^{1/2},$$

where k and m are projections of the angular momentum on the z-axes in the LF and MF, respectively. Unlike symmetric-top ions, such as H_3^+ or linear ions, the rotational frame transformation is not evaluated purely analytically for the asymmetric-top ion D_2H^+. Rotational functions of the ion and neutral molecule are needed

to transform the scattering matrix from the body frame (in which the electron moves in the reference frame of the molecule and in which the S-matrix is computed ab initio) to the lab frame (in which the electron moves relative to that lab frame). Rotational functions for the ion and the neutral molecule are obtained in a similar way, finding eigenvectors of the above Hamiltonian numerically. Rotational functions obtained by the diagonalization of the Hamiltonian are linear combinations of symmetric-top rotational functions

$$|N, m, p_1^{(rot)}, p_2^{(rot)}, j\rangle = \sum_k C_k \mathcal{R}^N_{km}(\alpha, \beta, \gamma) \tag{S11}$$

and are characterized by the following quantum numbers: N, m, the eigenvalue (parity) $p_1^{(rot)} = \pm 1$ with respect to E^*, and the eigenvalue with respect to (12), $p_2^{(rot)} = \pm 1$. The number j enumerates rotational states with otherwise identical quantum numbers. Because each symmetric-top function has well-defined quantum numbers p_1 and p_2 (see Supplementary Table 2)

$$\begin{aligned} E^*\mathcal{R}^N_{km} &= R^{\pi}_Z \mathcal{R}^N_{km} = (-1)^k \mathcal{R}^N_{km}, \\ (12)\mathcal{R}^N_{km} &= R^{\pi}_X \mathcal{R}^N_{km} = (-1)^N \mathcal{R}^N_{-km}, \\ (12)^*\mathcal{R}^N_{km} &= R^{\pi}_Y \mathcal{R}^N_{km} = (-1)^{N+k} \mathcal{R}^N_{-km}, \end{aligned} \tag{S12}$$

the expansion of Eq. (S11) includes only symmetric-top functions with $p_1^{(rot)} = p_1$, $p_2^{(rot)} = p_2$. In the approach used in Ref. [13], only the leading term in the expansion of Eq. (S11) was used.

2.4 Electronic wave functions

Spherical harmonics used for electronic part of the channel functions are linear combinations of C_{2v} irreps. Here, we give the linear combinations of the spherical harmonics having well-defined irreps Γ of C_{2v}:

$$\begin{aligned} \Gamma = A_1 : Y_{1,X}(\theta, \phi) &= \frac{1}{\sqrt{2}} \left[Y_{1,1}(\theta, \phi) + (-1)^1 Y_{1,-1}(\theta, \phi) \right], \\ \Gamma = B_1 : Y_{1,Z}(\theta, \phi) &= Y_{1,0}(\theta, \phi), \\ \Gamma = B_2 : Y_{1,Y}(\theta, \phi) &= \frac{1}{i\sqrt{2}} \left[Y_{1,1}(\theta, \phi) - (-1)^1 Y_{1,-1}(\theta, \phi) \right]. \end{aligned} \tag{S13}$$

The inversion operator E^* acts on the functions as

$$E^* Y_{1,\lambda}(\theta', \phi') = (-1)^1 Y_{1,\lambda}(\theta', \phi').$$

Ion	Deuteron pair nuclear spin	Symmetry	Multiplicity
D_2H^+	$I_D = 0$	A_1	1
	$I_D = 1$	B_2	3
	$I_D = 2$	A_1	5

Supplementary Table 4: Irreducible representations of nuclear spin states for different deuteron pair nuclear spins I_D for D_2H^+ ions.

The operator (12) acts on functions of Eq. (S13) according to the rules given in Supplementary Table 1 and Supplementary Table 2.

2.5 Nuclear spin wave functions

Similarly to the vibrational functions, the nuclear-spin functions $\Phi_{\rm ns}$ are unchanged after E^*

$$E^*\Phi_{\rm ns} = \Phi_{\rm ns} \tag{S14}$$

and, therefore, can only be of the A_1 and B_2 irreps. Supplementary Table 4 (from Ref. [15]) summarizes allowed irreps for different values of the deuteron pair nuclear spin I_D of the D_2H^+ ions.

3 Kinetic temperature DR rate coefficient for D_2H^+

To facilitate the use of our data in astrochemical models, we have converted our experimental merged-beams DR rate coefficients $\alpha^{\rm mb}(E_{\rm d})$ into kinetic temperature thermal rate coefficients $\alpha^{\rm k}(T_{\rm k})$, where $T_{\rm k}$ is the kinetic temperature of the gas. The two-step conversion procedure is the same as described in our previous studies [17–19].

First, the merged-beams DR rate coefficient is converted into a cross section $\sigma(E)$ that depends on the collision energy E, using the known merged-beams collision energy distribution $f_{\rm mb}(E; E_{\rm d})$, which is a function of the detuning energy $E_{\rm d}$. The procedure is explained in detail in [20]. In short, we make use of the relation

$$\alpha^{\rm mb}(E_{\rm d}) = \int_0^\infty \sigma(E)\sqrt{\frac{2E}{m_{\rm e}}} f_{\rm mb}(E; E_{\rm d}) dE, \tag{S15}$$

where $m_{\rm e}$ is the electron mass. The cross sections are derived iteratively, by creating a discrete generic cross section histogram and varying it until the application of Eq. S15 accurately reproduces the measured merged-beams rate coefficients.

In the second step the DR cross sections are convolved with Maxwell-Boltzmann distributions for various

kinetic temperatures T_k, applying

$$\alpha^\mathrm{k}(T_\mathrm{k}) = \int_0^\infty \sigma(E)\sqrt{\frac{2E}{m_\mathrm{e}}}\sqrt{\frac{4E}{\pi(k_\mathrm{B}T_\mathrm{k})^3}}e^{-E/k_\mathrm{B}T_\mathrm{k}}dE, \tag{S16}$$

where k_B is the Boltzmann constant.

Figure 3**c** in the main manuscript shows the kinetic temperature rate coefficient for D_2H^+ as a function of the gas kinetic temperature. The uncertainties of the measurement are propagated through the procedure and displayed in the plot by grey error bands.

To support the inclusion of the kinetic temperature rate coefficients in astrochemical databases, we provide two analytical representations of the rate coefficient and its error bands. The first fit function, as employed for DR in [20], is a continuously differentiable function that is optimized to account for resonant DR features (i.e. peaks and troughs), given by

$$\alpha^\mathrm{k}(T_\mathrm{k})[\mathrm{cm}^3\mathrm{s}^{-1}] = A\left(\frac{300}{T_\mathrm{k}[\mathrm{K}]}\right)^n + T_\mathrm{k}[\mathrm{K}]^{-1.5}\sum_{r=1}^{8} c_r \exp\left(-\frac{T_r}{T_\mathrm{k}[\mathrm{K}]}\right), \tag{S17}$$

where A, n, c_r and T_r are fit parameters, the values of which are given in Supplementary Table 5. The relative deviation of the fit does not exceed 0.05% .

The second representation of the thermal rates utilizes Arrhenius–Kooij (AK) functions, commonly employed in astrochemistry, combustion chemistry, and related chemical databases. The application of a single AK function alone is insufficient to accurately model the kinetic rate coefficient across the entire temperature range. To that end, in line with the approach of [18], we use a set of piecewise-joined AK fit functions over selected temperature intervals

$$\alpha^\mathrm{k}(T_\mathrm{k})[\mathrm{cm}^3\mathrm{s}^{-1}] = A_i\left(\frac{T_\mathrm{k}[\mathrm{K}]}{300}\right)^{\beta_i} e^{-\frac{\gamma_i}{T_\mathrm{k}[\mathrm{K}]}}, \tag{S18}$$

where A_i, β_i, and γ_i are the fit parameters for various temperatures, listed in Supplementary Table 6. The relative deviation of the fit does not exceed 7.8%. In accordance with KIDA conventions [21] we provide the uncertainty of α^k by means of the log-normal factor $F = \exp(\Delta\alpha^\mathrm{k}/\alpha^\mathrm{k})$. The value of $F(T_\mathrm{k})$ is determined by averaging the upper and lower uncertainty bands. Subsequently, fitting is performed over the same temperature intervals as defined for the AK functions, by employing the following fit function

$$F(T_\mathrm{k}) = F_{0,i}\exp\left(g_i\left(\frac{1}{T_\mathrm{k}[\mathrm{K}]} - \frac{1}{300}\right)\right), \tag{S19}$$

where $F_{0,i}$ and g_i denote the fit parameters, provided in Supplementary Table 6 .

$$F_{0,i} = F_{0,0} \exp\left(\frac{g_i - g_0}{300}\right) \prod_{j=1}^{i} \exp\left(\frac{g_{j-1} - g_j}{T_j}\right). \qquad \text{(S20)}$$

To facilitate the implementation of plasma kinetic rate coefficients in models of astronomical environments or technical plasma, it is often beneficial to use continuous functions. However, equations S18 and S19 do not ensure continuous behavior of the functions at the boundaries of the temperature intervals. Therefore, we imposed the following dependencies on parameters $F_{0,i}$ and A_i

$$A_i = A_0 \prod_{j=1}^{i} \left(\frac{T_j}{300}\right)^{\beta_{j-1}-\beta_j} e^{\frac{\gamma_j - \gamma_{j-1}}{T_j}}, \qquad \text{(S21)}$$

$$F_{0,i} = F_{0,0} \exp\left(\frac{g_i - g_0}{300}\right) \prod_{j=1}^{i} \exp\left(\frac{g_{j-1} - g_j}{T_j}\right). \qquad \text{(S22)}$$

Parameter	Rate coefficient	Lower error limit	Upper error limit
A	2.61×10^{-8}	2.07×10^{-8}	3.26×10^{-8}
n	0.947	0.927	0.993
c_1	-1.66×10^{-5}	-1.20×10^{-5}	-2.58×10^{-5}
c_2	1.72×10^{-4}	1.24×10^{-4}	2.49×10^{-4}
c_3	4.34×10^{-4}	3.16×10^{-4}	6.05×10^{-4}
c_4	8.84×10^{-4}	6.78×10^{-4}	1.12×10^{-3}
c_5	9.78×10^{-4}	8.24×10^{-4}	1.05×10^{-3}
c_6	1.53×10^{-1}	1.23×10^{-1}	1.92×10^{-1}
c_7	-4.54×10^{-4}	-6.84×10^{-4}	1.00×10^{-3}
c_8	9.58×10^{-1}	7.74×10^{-1}	1.19
T_1	5.3	5.1	5.3
T_2	308	298	331
T_3	650	614	721
T_4	1380	1302	1517
T_5	2539	2473	2616
T_6	86670	86018	89061
T_7	10578	11568	14761
T_8	155272	156029	157027

Supplementary Table 5: Fit parameters for the kinetic temperature rate coefficient of D_2H^+ with its lower and upper error bounds, based on Eq. S17 as the fit function.

	Temperature range (K)			
Parameter	10–100	100–1000	1000–10000	10000–40000
A_i	3.15×10^{-8}	4.79×10^{-8}	2.17×10^{-7}	1.04×10^{-16}
β_i	-0.630	-0.389	-1.193	3.357
γ_i	-2.424	13.08	554.4	-54458
$F_{0,i}$	1.249	1.221	1.053	2.709
g_i	1.107	4.509	-59.07	233.2

Supplementary Table 6: Fit parameters for the D_2H^+ kinetic temperature rate coefficient and its relative uncertainty obtained using Eq. S18 and S19 as the fit functions.

References

[1] Z. Mašín, J. Benda, J. D. Gorfinkiel, A. G. Harvey, and J. Tennyson, *UKRmol+: a suite for modelling of electronic processes in molecules interacting with electrons, positrons and photons using the R-matrix method*, Comput. Phys. Comms. **249**, 107092 (2020).

[2] B. Cooper, M. Tudorovskaya, S. Mohr, A. O'Hare, M. Hanicinec, A. Dzarasova, J. Gorfinkiel, J. Benda, Z. Mašín, A. Al-Refaie, P. J. Knowles, and J. Tennyson, *Quantemol Electron Collision: an expert system for performing UKRmol+ electron molecule collision calculations*, Atoms **7**, 97 (2019).

[3] N. Sanna and F. A. Gianturco, *Differential cross sections for electron/positron scattering from polyatomic molecules*, Comput. Phys. Commun. **114**, 142–167 (1998).

[4] A. Jain and D. G. Thompson, *A program to generate the symmetry-Adapted rotational eigenfunctions and energy levels for asymmetric top molecules*, Comput. Phys. Commun. **35**, C–918 (1984).

[5] S. Chandra, *Remarks on the Test-Run Output by Jain and Thompson.*, Astrophys. Space Sci. **138**, 221–226 (1987).

[6] I. Rabadán, B. K. Sarpal, and J. Tennyson, *On the calculation of electron-impact rotational excitation cross sections for molecular ions*, J. Phys. B: At. Mol. Opt. Phys. **31**, 2077–2090 (1998).

[7] A. Faure and J. Tennyson, *Electron-impact rotational excitation of linear molecular ions*, Mon. Not. R. Astron. Soc. **325**, 443–448 (2001).

[8] S.-I. Chu and A. Dalgarno, *Rotational excitation of CH^+ by electron impact*, Phys. Rev. A **10**, 788–792 (1974).

[9] D. A. Neufeld and A. Dalgarno, *Electron-impact excitation of molecular ions*, Phys. Rev. A **40**, 633–637 (1989).

[10] A. Faure, V. Kokoouline, C. H. Greene, and J. Tennyson, *Near-threshold rotational excitation of molecular ions by electron impact*, J. Phys. B: At. Mol. Phys. **39**, 4261–4273 (2006).

[11] A. Kálosi, M. Grieser, R. von Hahn, U. Hechtfischer, C. Krantz, H. Kreckel, D. Müll, D. Paul, D. W. Savin, P. Wilhelm, A. Wolf, and O. Novotný, *Laser probing of the rotational cooling of molecular ions by electron collisions*, Phys. Rev. Lett. **128**, 183402 (2022).

[12] J. Forer, D. Hvizdoš, M. Ayouz, C. H. Greene, and V. Kokoouline, *Kinetic rate coefficients for electron-driven collisions with CH^+: dissociative recombination and rovibronic excitation*, Mon. Not. R. Astron. Soc. **527**, 5238–5243 (2024).

[13] V. Kokoouline and C. H. Greene, *Theoretical study of dissociative recombination of C_{2v} triatomic ions: Application to H_2D^+ and D_2H^+*, Phys. Rev. A **72**, 022712 (2005).

[14] P. R. Bunker and P. Jensen, *Molecular Symmetry and Spectroscopy* (NRC Research Press, 1998).

[15] V. Kokoouline and C. H. Greene, *Triatomic dissociative recombination theory: Jahn-Teller coupling among infinitely many Born-Oppenheimer surfaces*, Faraday Discuss. **127**, 413 (2004).

[16] B. R. Johnson, *On hyperspherical coordinates and mapping the internal configurations of a three body system*, J. Chem. Phys. **73**, 5051 (1980).

[17] O. Novotný, P. Wilhelm, D. Paul, Á. Kálosi, S. Saurabh, A. Becker, K. Blaum, S. George, J. Göck, M. Grieser, F. Grussie, R. von Hahn, C. Krantz, H. Kreckel, C. Meyer, P. M. Mishra, D. Muell, F. Nuesslein, D. A. Orlov, M. Rimmler, V. C. Schmidt, A. Shornikov, A. S. Terekhov, S. Vogel, D. Zajfman, and A. Wolf, *Quantum-state-selective electron recombination studies suggest enhanced abundance of primordial HeH^+*, Science **365**, 676–679 (2019).

[18] D. Paul, M. Grieser, F. Grussie, R. von Hahn, L. W. Isberner, Ábel Kálosi, C. Krantz, H. Kreckel, D. Müll, D. A. Neufeld, D. W. Savin, S. Schippers, P. Wilhelm, A. Wolf, M. G. Wolfire, and O. Novotný, *Experimental Determination of the Dissociative Recombination Rate Coefficient for Rotationally Cold CH^+ and Its Implications for Diffuse Cloud Chemistry*, Astrophys. J. **939**, 122 (2022).

[19] Á. Kálosi, L. Gamer, M. Grieser, R. von Hahn, L. W. Isberner, J. I. Jäger, H. Kreckel, D. A. Neufeld, D. Paul, D. W. Savin, *et al.*, *Dissociative recombination of rotationally cold OH^+ and its implications for the cosmic ray ionization rate in diffuse clouds*, Astrophys. J. **955**, L26 (2023).

[20] O. Novotný, A. Becker, H. Buhr, C. Domesle, W. Geppert, M. Grieser, C. Krantz, H. Kreckel, R. Repnow, D. Schwalm, K. Spruck, J. Stützel, B. Yang, A. Wolf, and D. W. Savin, *Dissociative Recombination Measurements of HCl^+ Using an Ion Storage Ring*, Astrophys. J. **777**, 54 (2013).

[21] V. Wakelam, E. Herbst, J.-C. Loison, I. W. M. Smith, V. Chandrasekaran, B. Pavone, N. G. Adams, M.-C. Bacchus-Montabonel, A. Bergeat, K. Béroff, V. M. Bierbaum, M. Chabot, A. Dalgarno, E. F. van Dishoeck, A. Faure, W. D. Geppert, D. Gerlich, D. Galli, E. Hébrard, F. Hersant, K. M. Hickson, P. Honvault, S. J. Klippenstein, S. L. Picard, G. Nyman, P. Pernot, S. Schlemmer, F. Selsis, I. R. Sims, D. Talbi, J. Tennyson, J. Troe, R. Wester, and L. Wiesenfeld, *A kinetic database for astrochemistry (KIDA)*, Astrophys. J. Suppl. S. **199**, 21 (2012).
